# DAMPs prognostic signature predicts tumor immunotherapy, and identifies immunosuppressive mechanism of pannexin 1 channels in pancreatic ductal adenocarcinoma

**DOI:** 10.3389/fimmu.2024.1516457

**Published:** 2025-01-15

**Authors:** Qianxue Wu, Qian Xiao, Xin Tang, Liuying Li, Daqiang Song, Yang Zhou, Benhua Li, Guosheng Ren, Fang Luo

**Affiliations:** ^1^ Department of Hepatobiliary Surgery, The First Affiliated Hospital of Chongqing Medical University, Chongqing, China; ^2^ Department of Breast and Thyroid Surgery, Women and Children’s Hospital of Chongqing Medical University, Chongqing, China; ^3^ Chongqing Key Laboratory of Molecular Oncology and Epigenetics, The First Affiliated Hospital of Chongqing Medical University, Chongqing, China; ^4^ Department of Respiratory and Critical Care Medicine, The First People’s Hospital of Chongqing Liang Jiang New Area, Chongqing, China; ^5^ Department of Clinical Laboratory, The Second People’ s Hospital of Liangshan yi Autonomous Prefecture, Xichang, China; ^6^ Clinical Molecular Medicine Testing Center, The First Affiliated Hospital of Chongqing Medical University, Chongqing, China; ^7^ Department of Breast and Thyroid Surgery, The First Affiliated Hospital of Chongqing Medical University, Chongqing, China

**Keywords:** PANX1, DAMPs, PDAC, NOD1, NFκB

## Abstract

**Background:**

Damage-associated molecular patterns (DAMPs) induced by immunogenic cell death (ICD) may be useful for the immunotherapy to patients undergoing pancreatic ductal adenocarcinoma (PDAC). The aim of this study is to predict the prognosis and immunotherapy responsiveness of PDAC patients using DAMPs-related genes.

**Methods:**

K-means analysis was used to identify the DAMPs-related subtypes of 175 PDAC cases. The significance of gene mutation and immune status in different subtypes was detected. LASSO regression was used to construct a DAMPs-related prognostic signature to predict the immunotherapy responsiveness of PDAC. Subsequently, *in vivo* and *in vitro* experiments and Bulk-RNA seq were used to verify the effect of hub gene pannexin 1 (PANX1) on PDAC.

**Results:**

Two subtypes were clustered based on the expression levels of DAMPs genes from 175 PDAC patients. Besides, the prognosis and immune landscape in up-regulated DAMPs expression subtypes was poor. In addition, we constructed a DAMPs-related prognostic signature that correlated with immune cell infiltration and predicted immunotherapy or chemotherapy responsiveness of patients with PDAC. Mechanically, through Bulk-RNA sequencing and experiments, we found that PANX1 promoted tumor progression and immune regulation via the ATP release to active NOD1/NFκB signaling pathway in PDAC.

**Conclusion:**

Our in silico analyses established a classification system based on ICD-related DAMPs genes in PDAC, and constructed a DAMPs-related prognostic model to predict the efficacy of immunotherapy. This study will provide a new perspective for targeting the DAMPs-related molecule PANX1 in the treatment of PDAC.

## 1 Introduction

Pancreatic ductal adenocarcinoma (PDAC) is a malignant tumor which emerges as concealed incidence, rapid progress, poor treatment effect and prognosis. The overall survival (OS) of five-year is less than 10%, and its global incidence is on the rise in PDAC (1). The tumor microenvironment (TME) of PDAC may lead a poor efficacy of single immune checkpoint inhibitors (ICIs) in the treatment of PDAC (2–4). Previous study has shown the combination of therapy strategies for reversing the immunosuppressive state of TME and responding to ICIs in PDAC (5). Therefore, ICIs combined with targeting PDAC TME are likely to be an effective strategy to improve the responsiveness of PDAC immunotherapy. Recently, studies have confirmed that induced-immunogenic cell death (ICD) sensitized PDAC cells to ICIs (6), and further prompted biomarkers of ICD may be candidate prognostic factors for immunotherapy (7, 8).

ICD includes any type of cell death caused by immune response, accidental cell death or regulatory cell death caused by activation of immune response (9). The secretion of damage-related molecular patterns (DAMPs) is a prominent feature of ICD (10). Calreticulin (CALR) is one of the DAMPs molecules, located in the endoplasmic reticulum lumen. After ICD induction, CALR was translocated to the surface of dead cells and attracted special antigen presenting cells (APC) as a “eating me” signal (11). Heat shock proteins (HSPs) are also translocated to the plasma membrane under stress conditions and bind to APC surface receptors CD91 and CD40 to promote the cross-presentation of tumor antigens on MHC-I molecules, subsequently induced CD8+ T-cell responses (12). High mobility group protein B1 (HMGB1) and adenosine triphosphate (ATP) are also important DAMPs secreted by ICD. Among them, HMGB1 is considered to be a marker of late ICD, which bind to Toll-like receptors (TLR) 2 and 4 and other pattern recognition receptors expressed on APCs (13). TNFα promotes pannexin 1 (PANX1) cleavage through a caspase8/3-dependent pathway to enhance the immunogenicity of cancer cells, leading to dendritic cell maturation and T cell activation, which is a key determinant of chemotherapy-induced antitumor immunity (14). PANX1-mediated eATP export regulates effector and memory CD8+ T cells through distinct purinergic receptors and distinct metabolic and signaling pathways. Due to DAMPs and their receptors stimulated positive immune responses in TME, targeting DAMPs and their receptors have the potential to treat PDAC.

**Figure 1:**
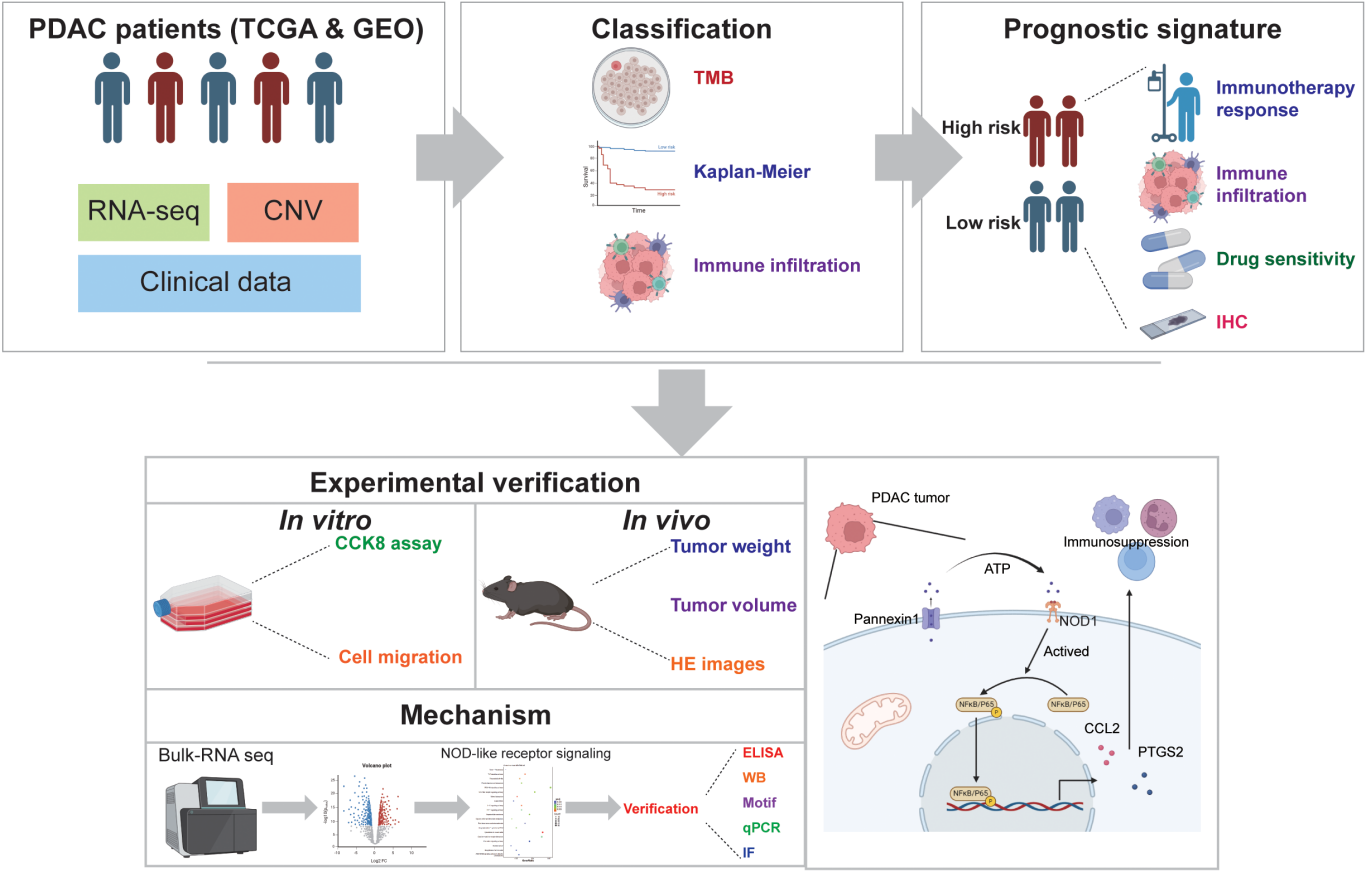
Flow chart of the data analysis process.

In this study, as shown in **Figure 1**, we comprehensively analyzed the characteristics of DAMPs genes in PDAC to explore the role in TME and survival in patients with PDAC. Furthermore, we constructed a signature based on DAMPs-related genes to predict survival and treatment of patients with PDAC. We have uncovered a distinct function of the key molecule PANX1 in PDAC. PANX1 was found to stimulate the expression of PTGS2 and CCL2 via the ATP release to active NOD1/NFκB signaling pathway, influencing immune infiltration and facilitating the progression of PDAC. Our findings may provide new clues to explore the potential molecular mechanisms of different responsiveness of PDAC patients to immunotherapy.

## 2 Methods and materials

### 2.1 Patient data acquisition

The RNA-seq expression profile and single nucleotide mutation data of 179 cases of PDAC samples were obtained from The Cancer Genome Atlas (TCGA) data portal (https://cancergenome.nih.gov/). The patients with incomplete clinical information were excluded, and the follow-up information of 175 patients was obtained. The RNA-seq expression profile (GSE57495) (15) of 63 PDAC samples were obtained from Gene Expression Omnibus (https://www.ncbi.nlm.nih.gov/geo/).

### 2.2 Consensus clustering

Thirty-two DAMPs-related genes were collected for PDAC consensus clustering (16). PDAC samples were classified by DAMPs-related gene expression and patient follow-up information. After univariate cox analysis, the ‘ConsensusClusterPlus’ R package was used for consensus clustering, and the PDAC samples were divided into up to 9 subtypes. According to the correlation of gene expression within the clustering, the best consensus clustering can be found.

### 2.3 Identification of differentially expressed genes and gene enrichment

DEGs between high expression group and low expression group of DAMPs-related gene were analyzed. The cut-off standard of differentially expressed genes was FDR (False discovery rate) corrected *p*-value< 0.001, | logFC | > 2. Up-regulated DEGs were used for enrichment analysis. “clusterProfiler” package, “org.Hs.eg.db” package, “enrichplot” package, “GOplot” package and “ggplot2” package of R software were used for GO analysis and KEGG analysis.

### 2.4 The relationship between DAMPs and tumor immune microenvironment

Immune and stromal cell scores were calculated after applying ESTIMATE algorithm in “estimate” and “limma” R-package to predict tumor purity and the presence of infiltrating matrix/immune cells in PDAC tissue. The content of immune cells, the expression levels of Human Leukocyte Antigen (HLA) family genes and immune checkpoint molecules between the two subtypes of DAMPs were compared. SsGSEA analysis was used to determine the correlation between tumor immune function (Type II IFN Response, Cytolytic activity, Inflammation-promoting, Type I IFN Response, APC co-inhibition, MHC class I, APC co-stimulation, CCR, Parainflammation, HLA, T cell co-inhibition, Check-point, and T cell co-stimulation) and DAMPs prognostic risk signature.

### 2.5 Construction of DAMPs prognostic risk signature

TCGA dataset and GSE57495 were used for train set and validation set, respectively. Univariate Cox regression model was used to identify DAMPs-related gene with prognostic value. The “glmnet” package was used to perform Least Absolute Shrinkage and Selection Operator (LASSO) of DAMPs-related gene related with prognosis. Multivariate Cox regression was used to identify DAMPs-related gene with independent prognostic value. The DAMPs-related gene prognostic signature was selected via the lowest Akaike Information Criterion (AIC). The risk score of each patient was calculated according to the following formula: $Risk\;score=\sum_{k=0}^{n}coef\left(k\right)*x\left(k\right)$ , where coef (k) and x (k) were regression coefficients. Kaplan-Meier plotter was drawn to compare OS or progression free survival (PFS) of PDAC patients with different prognostic risk scores. The receiver operating characteristic curve (ROC) curve was calculated using R packet “survivalROC” to calculate the prognostic value of DAMPs-related gene prognostic risk score for 1-year, 3-year, 5-year prediction of PDAC patients, as well as other clinical features (Age, Sex, Grade, Stage, T stage, and N stage).

### 2.6 Prediction of response to immunotherapy

The tumor mutation burden (TMB) score (mutation frequency) was calculated using the single nucleotide mutation data of TCGA. The TMB scores of high and low DAMPs prognostic risk groups were compared. Kaplan-Meier plotter was used to analyze the OS of PDAC patients with different TMB levels and DAMPs-related prognostic risk scores. The expression profile of PDAC samples was imported into TIDE platform (Tumor Immune Dysfunction and Exclusion, http://tide.dfci.harvard.edu/), and the TIDE score of each sample was calculated. The TIDE score was used to evaluate the response of patients with PDAC with different DAMPS-related prognostic risk scores to immunotherapy.

### 2.7 Drug sensitivity

The R packet “pRRophetic” was used to select potential sensitive drugs for PDAC based on DAMPs-related prognostic signature. Spearman correlation coefficient was used to evaluate the correlation between drug IC_50_ and DAMPs-related prognostic signature. The differences of drug IC_50_ between high and low DAMPs-related prognostic risk score groups were compared.

### 2.8 The human protein atlas and motif

The immunohistochemical staining of PANX1, TLR3, DDX58, P2RY2, and AGER in normal and PDAC tissues were obtained from the human protein atlas (www.proteinatlas.org/). JASPAR database (jaspar.elixir.no) was used to predict the sequence of transcription factors (NFκB) binding promoters (CCL2 and PTGS2), and those with high prediction scores were selected for plotting.

### 2.9 Constructs and cell transfection

The lentivirus shRNA sequence was as follows: Sh1: 5-CCCACCTTCGATGTTCTACAT-3; Sh2: 5- GCATGTATCTACTTGAGCTAT-3. The expression vector encoding PANX 1 was constructed by cloning the open reading frame (ORF) of the specified gene into the pcDNA3.1 vector. Transfection of HEK293T cells was carried out using a 4:3:1 ratio with co-transfection of packaging plasmids pSPAX2 and pMD2G. The supernatant was harvested after 48 h, and the target cells were subjected to a 48 h re-infection with polybrene. PANX1 knock down or overexpressed Stable expression in Panc02 cells was achieved through selection with puromycin.

### 2.10 Quantitative real-time PCR

Isolate total RNA from cellular samples. Utilize reverse transcriptase to synthesize cDNA from the extracted RNA. Assemble the qPCR reaction mixture, comprising the cDNA template, appropriate primers, qPCR Master Mix, and nuclease-free water. Transfer the mixture to qPCR reaction tubes. The sequences of the primers are listed below (**Supplementary Table S1**). Execute the qPCR protocol on a thermocycler with the following thermal profile: initial enzyme activation at 95°C for 3 minutes, followed by 40 cycles of denaturation at 95°C for 15 seconds and annealing/extension at 60°C for 1 minute. Analyze the resulting Ct values using qPCR analysis software to conduct relative quantification. Normalize the expression levels to GAPDH as an endogenous control, with the formula for relative expression being 2^^(-ΔΔCt)^.

### 2.11 Western blot

Collect cell samples from each group, extract proteins, and determine protein concentrations using the BCA assay. Subject the protein samples to denaturation and separate them by electrophoresis. Transfer the separated proteins from the gel to a PVDF membrane. Block non-specific binding sites on the membrane with a blocking solution (5% skim milk). Add the primary antibody (Antibodies are listed below (**Supplementary Table S1**)) and incubate overnight at 4°C. The next day, wash the membrane with TBST buffer three times, for 5 minutes each. Add the secondary antibody. Wash the membrane three times, for 5 minutes each. Detect the signal from the secondary antibody using an ECL chemiluminescence detection system. Capture images and perform quantitative analysis using image analysis software.

### 2.12 Cell counting kit-8 and migration assay

Seed the cells to be tested (3×10^3^ cell) into a 96-well plate and culture them in a cell incubator. After culturing for a certain period, add 10 μl of CCK-8 reagent (C0038, Beyotime) to each well. Place the 96-well plate back into the cell incubator and continue to incubate for 1 h. Use a plate reader to measure the absorbance of each well at a wavelength of 450 nm. Seed the cells into a culture dish and allow them to grow until a monolayer is formed. When the cells reach an appropriate density, use a 200 μl pipette tip to create a straight line scratch in the dish. Gently wash away the cell debris at the scratch site with PBS. Add serum-free medium to facilitate cell migration. Observe the cell migration at 0 h and 24h under a microscope. Use image processing software to measure the change in scratch area over time.

### 2.13 Animal studies

Five to six-week-old male C57BL/6 mice were provided by Beijing Vital River Company. C57BL/6 mice were injected with 1 × 10^6^ PANX1 overexpressing or vehicle Panc02 cells into the groin of the mice. Tumor size was measured 1 time per five days using calipers. Tumor volume (mm^3^) was calculated using the formula: tumor volume = 0.5 × (L × W)^2^, where L represents the longest dimension, and W is the perpendicular dimension. This study was approved by the Experimental Animal Ethics Committee of The First Affiliated Hospital of Chongqing Medical University.

### 2.14 RNA seq and data analysis

Harvest cells from the PANX1-OE and Vehicle groups, and isolate total RNA utilizing Trizol reagent. Evaluate the RNA purity, integrity, and concentration with a bioanalyzer instrument. Post-cDNA library construction, proceed to sequencing using the Illumina platform. Once sequencing is complete, perform data quality control and sequence alignment to determine the expression levels of each gene. Subsequently, carry out differential gene expression analysis and functional annotation, including GO and KEGG enrichment analyses, to compare the gene expression profiles across the different groups.

### 2.15 ELISA, hematoxylin and eosin staining and immunofluorescence

ELISA was performed via procedure according to the manufacturer’s instructions. In brief, ATP levels were assessed using the ATP Content Assay Kit with Molybdophosphoric acid (Biosharp, Catalog Number: BL851A). Cellular samples were lysed similarly and centrifuged to collect the supernatant. Neutralized samples were reacted with assay reagents, incubated at 37°C, and the absorbance at 700 nm was measured. ATP concentrations were calculated from a standard curve. The sample concentrations were then calculated. The samples were stained first in a solution of hematoxylin, followed by eosin staining. Subsequent dehydration of the samples was carried out. Finally, cleaning, dehydration, and slide mounting were performed. The stained samples were then observed under a microscope to evaluate the staining effects and tissue structures. Tissue samples were fixed using paraformaldehyde. Goat serum was employed to block nonspecific binding. The samples were then incubated with primary antibody overnight at 4°C. Subsequently, washing was performed three times with PBST buffer. Next, fluorescent-labeled secondary antibody was added to bind with the primary antibody, followed by three additional washes. The samples were mounted on slides for microscopic observation. Fluorescence expression of target structures was examined and analyzed under a fluorescence microscope.

### 2.16 Statistical analysis

All data were analyzed by R software (version 4.0.3, https://www.r-project.org/). The Student’s t-test or one-way analysis of variance (ANOVA) was used to conduct differential comparisons between two or multiple groups, respectively. The method of non-parametric test uses wilcox.test for comparison and kruskal.test for multi-group global comparison. P< 0.05 was considered to be statistically significant.

## 3 Results

### 3.1 Consensus clustering identified two DAMPs-related subtypes

**Figure 2:**
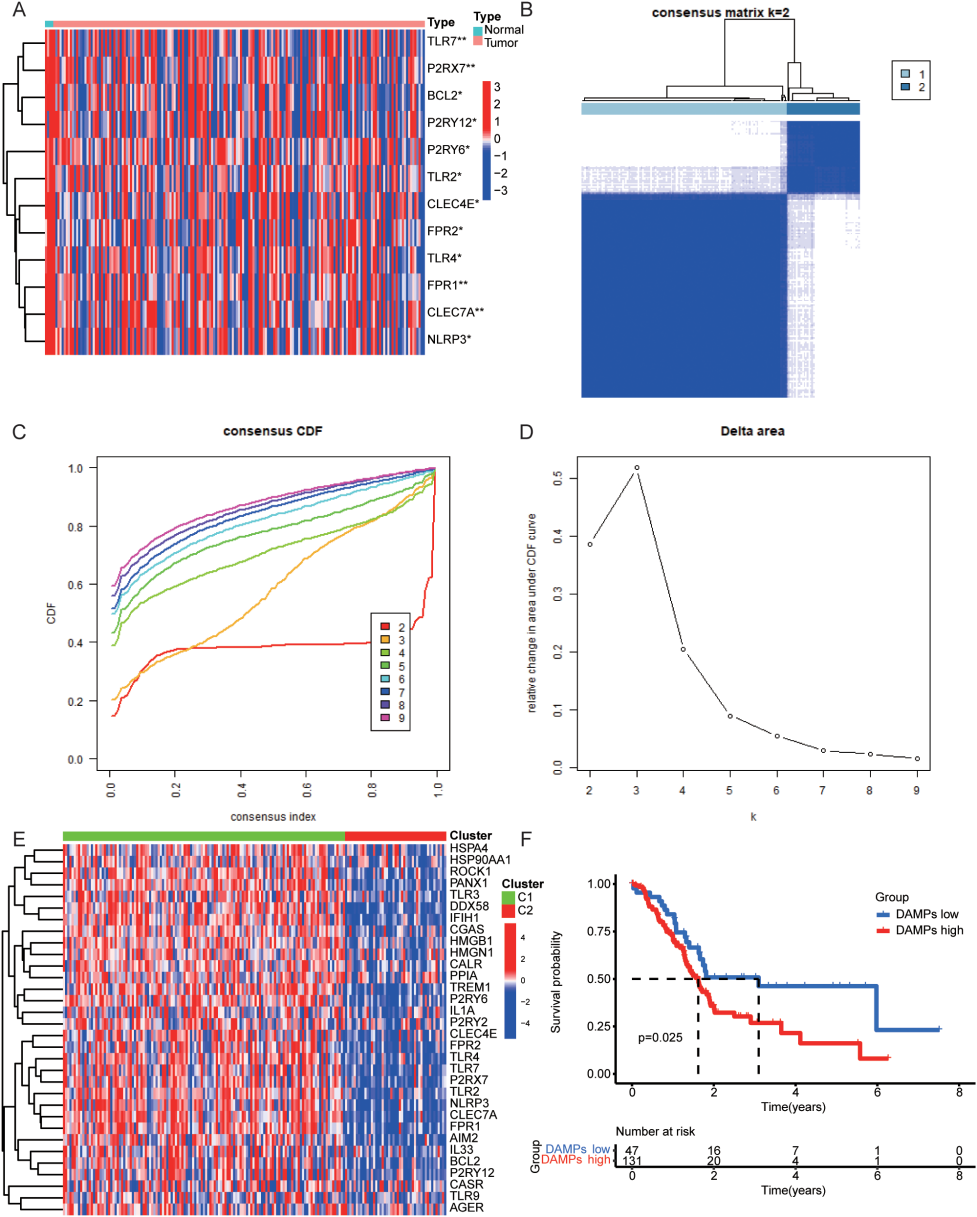
Consensus Clustering in PDAC. **(A)** Heatmap of the expression levels of DAMPs genes in normal and tumor samples of PDAC. **(B–D)** The best DAMPs clustering divides PDAC samples into two categories. **(E)** The differential expression of DAMPs-related genes between two DAMPs subtypes of PDAC. **(F)** OS of PDAC patients in different DAMPs subtypes.

First of all, we found some DAMPs-related genes were down-regulated in PDAC samples (**Figure 2A**) and their protein-protein interactions were visualized (**Supplementary Figure S1**). We subsequently utilized the expression of DAMPs-related genes and survival follow-up data for clustering. The results revealed the optimal classification of PDAC samples into two distinct subtypes. (**Figures 2B–D**). DAMPs-related genes were up-regulated in C1 and down-regulated in C2 (**Figure 2E**). In addition, we found that PDAC patients with high risk (C1) had a shorter OS (**Figure 2F**). Finally, we identified the DEGs for DAMPs subtypes in the whole genome (**Supplementary Figures S2A, B**). GO enrichment analysis showed that up-regulated DEGs were related to immune functions such as immunoglobulin production, immunoglobulin complex, antigen binding (**Supplementary Figures S2C, D**). KEGG enrichment analysis also showed that up-regulated DEGs were associated with Cytokine-cytokine receptor interaction, JAK-STAT signaling pathway, primary immunodeficiency and other signal pathways (**Supplementary Figure S2E**).

### 3.2 Somatic mutations and TME landscape in DAMPs subtypes

**Figure 3:**
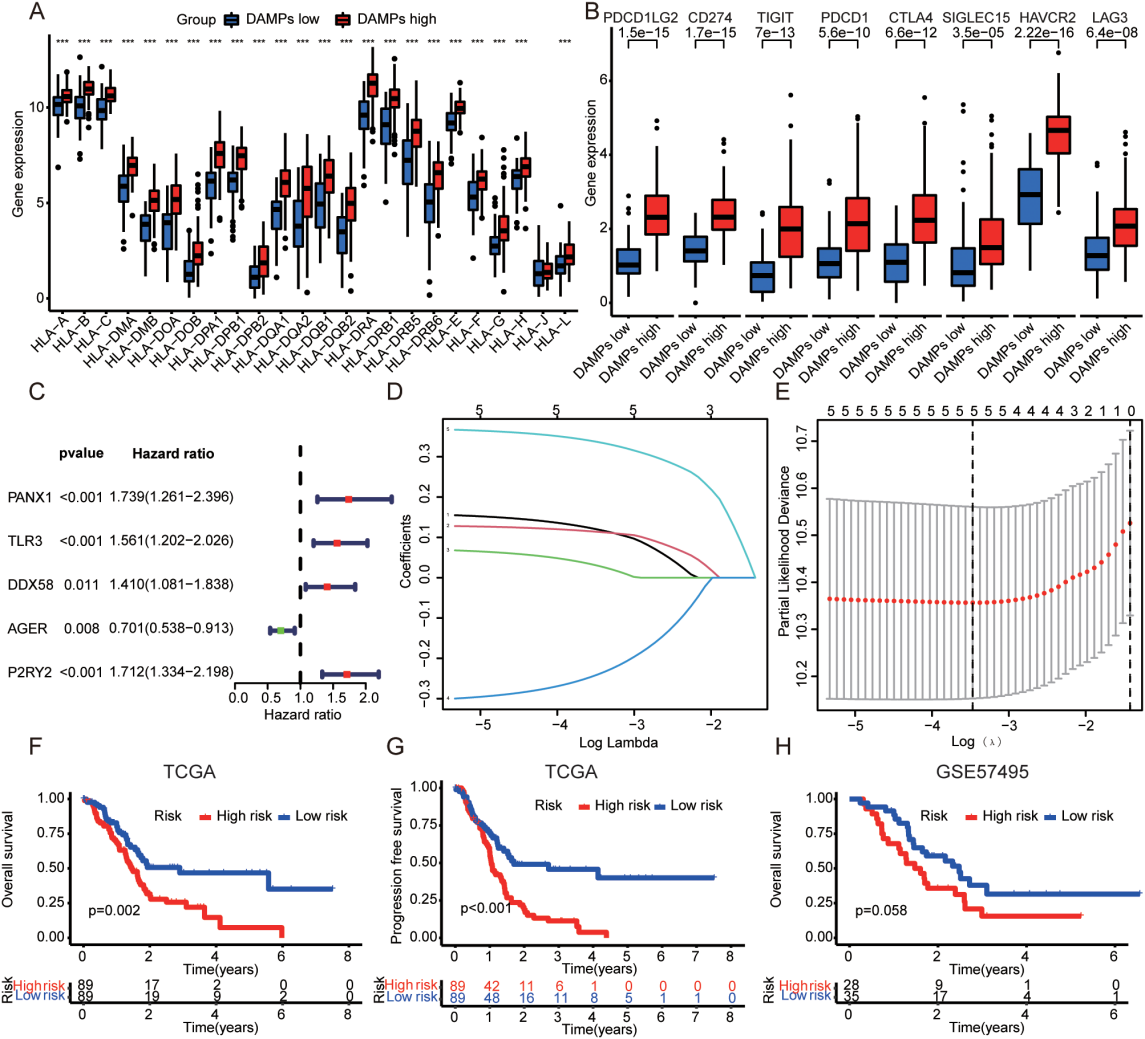
Construction of DAMPs prognostic signature. **(A)** Relationship between DAMPs subtypes and HLA family genes in PDAC. **(B)** Correlation between DAMPs subtypes and immune checkpoint molecules in PDAC. **(C)** Univariate cox analysis of DAMPs-related genes in PDAC. **(D)** Plots of the ten-fold cross-validation error rates. **(E)** LASSO coefficient profiles of the DAMPs-related genes. **(F, G)** The OS and PFS of PDAC patients with different prognostic risk score in TCGA dataset (train set). **(H)** The OS of PDAC patients with different prognostic risk score in GSE57495 dataset (validation set). ***P <0.001.

Next. We analyzed the mutation frequency of two DAMPs subtypes. The results showed that tumor mutation frequency of high-risk group was higher (**Supplementary Figure S3A**), including KRAS (64%), TP53 (66%), SMAD4 (21%), CDKN2A (17%) and TTN (10%). The mutation frequency of low-risk group was lower, including KRAS (53%), TP53 (31%), SMAD4 (24%), CDKN2A (20%) and TTN (18%). Then, the correlation between DAMPs subtypes and TME was analyzed. The results showed that the high-risk group was positively correlated with ESTMATES score, immune score and stromal score (**Supplementary Figures S3B, D**), and negatively correlated with TumorPurity (**Supplementary Figure S3E**). In order to evaluate the relationship between DAMPs subtypes and immune cells, the distribution of immune cells in each PDAC sample was analyzed. Among them, the content of Plasma cells in high-risk group decreased (**Supplementary Figure S3F**). In addition, HLA family genes were up-regulated in PDAC samples with high risk (**Figure 3A**). Finally, we found that immune checkpoint molecules were also up-regulated in PDAC with high risk (**Figure 3B**).

### 3.3 Construction and validation of DAMPs prognostic risk signature

**Figure 4:**
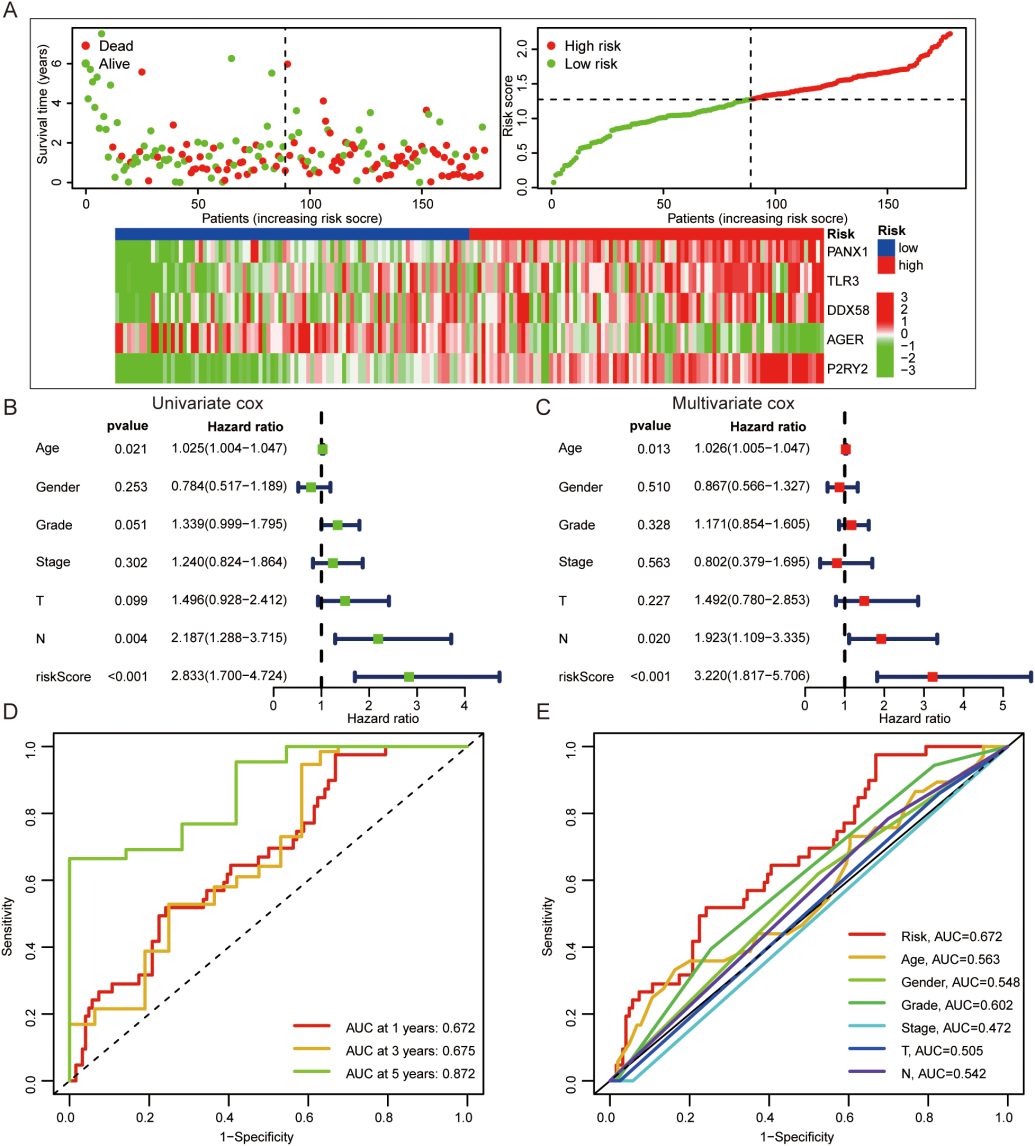
The prognostic value of DAMPs prognostic signature in PDAC. **(A)** The survival status of PDAC patients with different prognostic risk score and the gene expression level of DAMPs prognostic signature. **(B, C)** Univariate and multivariate cox regression of DAMPs prognostic risk score. **(D)** ROC curves of DAMPs prognostic risk score for PDAC patients at 1, 3, and 5 years. **(E)** ROC curves of DAMPs prognostic risk score, Age, Sex, Grade, Stage, T stage and N stage for PDAC patients.

In order to evaluate the prognostic value of DAMPs in PDAC, 32 DAMPs-related genes were used to construct a prognostic signature. Univariate cox was used to determine 9 DAMPs-related genes with prognostic value (**Figure 3C**). Next, a prognostic signature was constructed by LASSO regression (**Figures 3D, E**). Kaplan-Meier plotter showed that PDAC patients with high risk had a poor prognosis (**Figures 3F, G**). Interestingly, in the validated dataset GSE57495, PDAC patients with high risk also had a poor prognosis (p = 0.058, **Figure 3H**). PDAC patients with high risk had more death status (**Figure 4A**). Univariate and multivariate cox regression showed that DAMPs prognostic risk signature had independent prognostic value (**Figures 4B, C**). Next, the prognostic value of DAMPs prognostic risk signature was evaluated by ROC curve. The results showed that the 1-, 3- and 5-year AUC values of DAMPs prognostic risk signature for PDAC patients were 0.672, 0675, and 0.872, respectively (**Figure 4D**). The AUC value of DAMPs prognostic risk score was greater than other clinical prognostic characteristics (Age, Sex, Grade, Stage, T stage, and N stage) (**Figure 4E**). Finally, we found that the C index of the risk score was greater than 0.6 (**Supplementary Figure S4A**), and the 1-, 3-and 5-year survival rates of PDAC patients could be calculated based on clinical features and risk scores (**Supplementary Figures S4B, C**).

### 3.4 Relationship between DAMPs prognostic risk signature and immune cell infiltration

**Figure 5:**
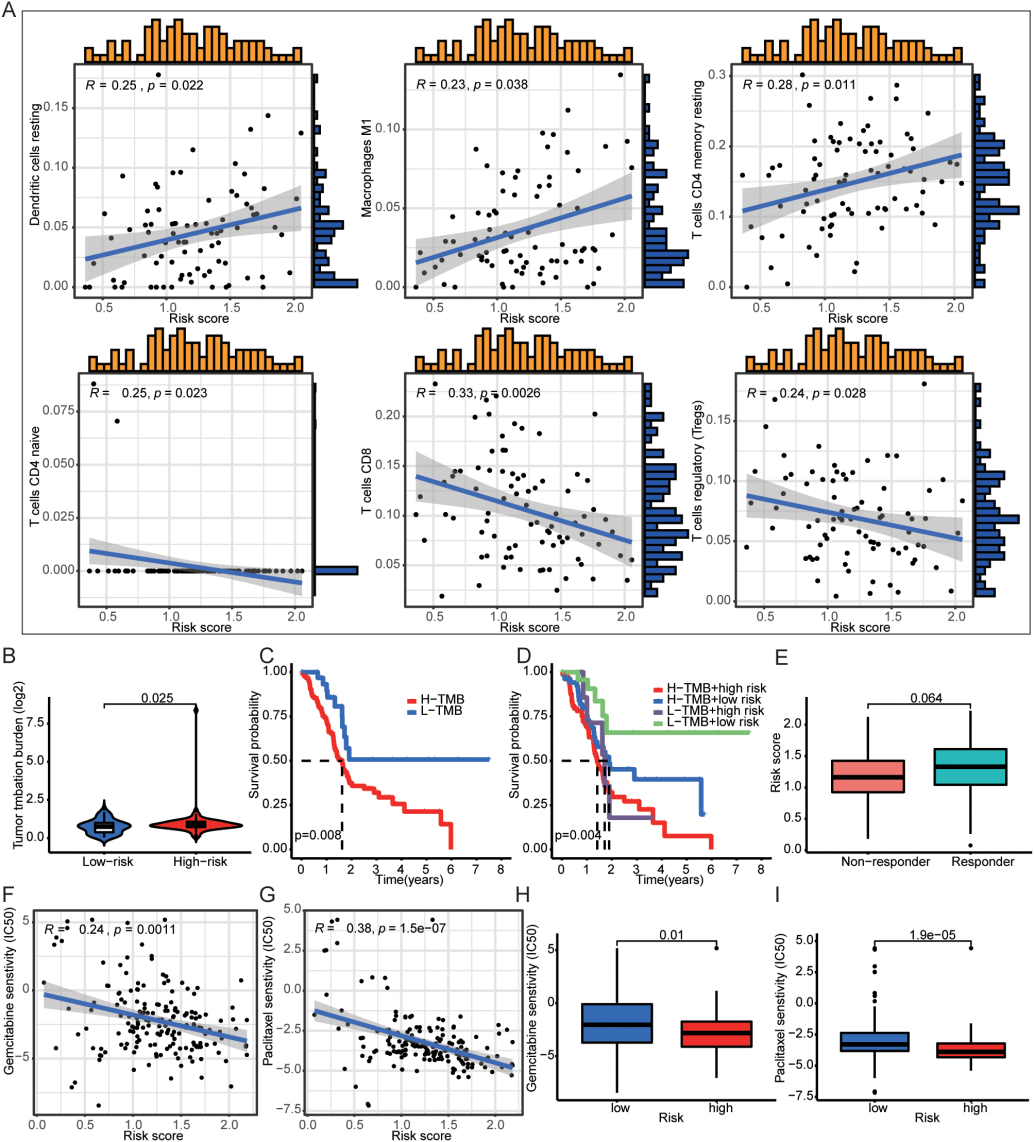
DAMPs prognostic signature was associated with immune cells infiltration. **(A)** The risk score was positively correlated with Dendritic cells resting, Macrophages M1, and T cells CD4 memory resting, and the risk score was negatively correlated with T cells CD4 naïve, T cells CD8, and T cells regulatory (Tregs). **(B)** PDAC patients with high DAMPs prognostic risk score had higher TMB. **(C, D)** Comprehensive survival analysis of patients with different tumor mutation loads and different prognostic risk. **(E)** The relationship between DAMPs prognostic score and tumor immunotherapy response. **(F–I)** PDAC patients with high prognostic risk are less sensitive to gemcitabine and paclitaxel.

Next, we found that the high-risk group of DAMPs signature was associated with tumor immunity, such as APC-co-inhibition (**Supplementary Figure S4D**). The risk score was positively correlated with Dendritic cells resting, Macrophages M1, and T cells CD4 memory resting. And the risk score was negatively correlated with T cells CD4 naïve, T cells CD8, and T cells regulatory (Tregs) (**Figure 5A**). Interestingly, PDAC with high risk had higher TMB score (**Figure 5B**). Not only the patients with high TMB score had poor prognosis (**Figure 5C**), but also the patients with high TMB score and high risk of DAMPs prognostic risk signature had the worst onset of prognosis (**Figure 5D**). Moreover, PDAC patients who responded to immunotherapy appeared to have a higher risk score (*p* = 0.064, **Figure 5E**).

### 3.5 Prediction of drug sensitivity by DAMPs prognostic risk signature

To investigate whether DAMPs prognostic risk signature could predict treatment benefit, we analyzed drug sensitivity screening. We found that the prognostic risk score of DAMPs was negatively correlated with IC50 of gemcitabine and paclitaxel (**Figures 5F, G**), and the IC50 of PDAC patients with high risk of was decreased (**Figures 5H, I**). The protein expression of PANX1, TLR3, DDX58, and P2RY2 were up-regulated, and AGER was down-regulated in PDAC tissues (**Supplementary Figure S5A**). However, TLR3, DDX58, and P2RY2 were not appropriated prognostic factors (**Supplementary Figures S5B–D**).

### 3.6 PANX1 promoted PDAC progression *in vitro and in vivo*

**Figure 6:**
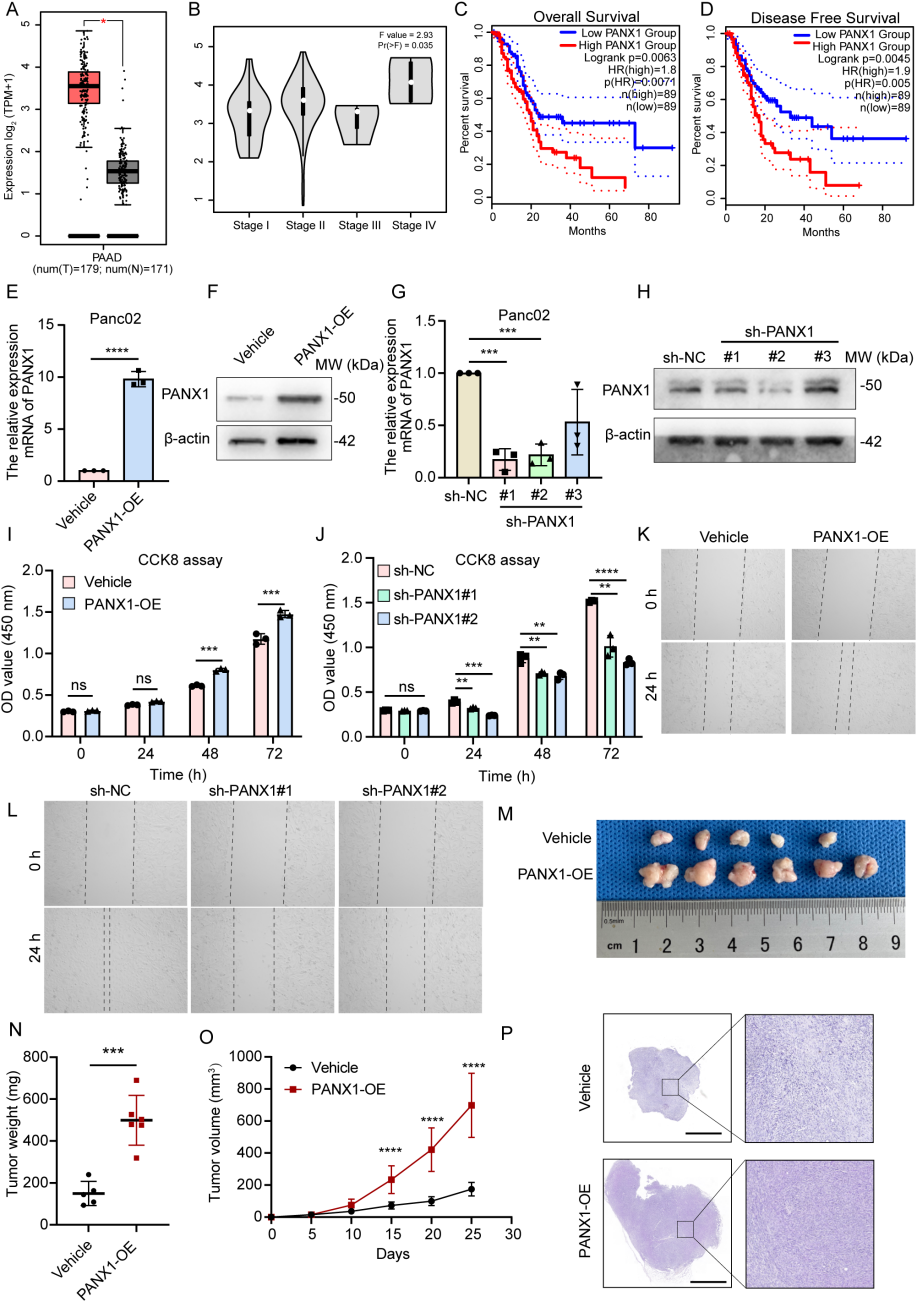
PANX1 is a poor prognostic factor for PDAC. **(A, B)** PANX1 was upregulated in PDAC cancer and high stage samples. **(C, D)** PANX1 was associated with poor OS and DFS. **(E, F)** PANX 1 was overexpressed in PDAC tumor cell line Panc02. **(G, H)** PANX 1 was knock-down in PDAC tumor cell line Panc02. **(I, J)** CCK8 assay showed overexpression of PANX 1 enhanced and knock-down of PANX 1 inhibited cell viability. **(K, L)** Overexpression of PANX 1 promoted and knock-down of PANX 1 inhibited cell migration. **(M)** The image of PANX1 promoting subcutaneous tumor growth in mice. **(N, O)** The tumor weight of PANX1-OE group and tumor growth curve was higher than that of Vehicle group. **(P)** The HE images of subcutaneous tumors in mice, scale bar = 2 mm. **P<0.01, ***P<0.001, ****P<0.0001, n.s, no significance.

However, PANX1 was upregulated in PDAC tumor tissue and advanced stage samples (**Figures 6A, B**). And PDAC patients with high expression of PANX1 had worse OS and DFS (**Figures 6C, D**). Therefore, to explore the key determinant of PANX1 in PDAC, we designed a series of experiments. PANX1 was overexpressed and knock-down in mouse PDAC cell line Panc02 (**Figures 6E–H**). Notably, the overexpression and knock-down of PANX1 significantly increased and inhibited cell viability (**Figures 6I, J**). Similarly, PANX1 also affected cell migration (**Figures 6K, L**). Mice subcutaneous transplantation model were established to evaluate the effect of PANX1 on tumor growth (**Figure 6M**). The injection of PANX1-OE Panc02 cell showed faster growth rates than the Vehicle group *in vivo* (**Figures 6N–P**).

### 3.7 Mechanism of PANX1-induced immunosuppression in PDAC

**Figure 7:**
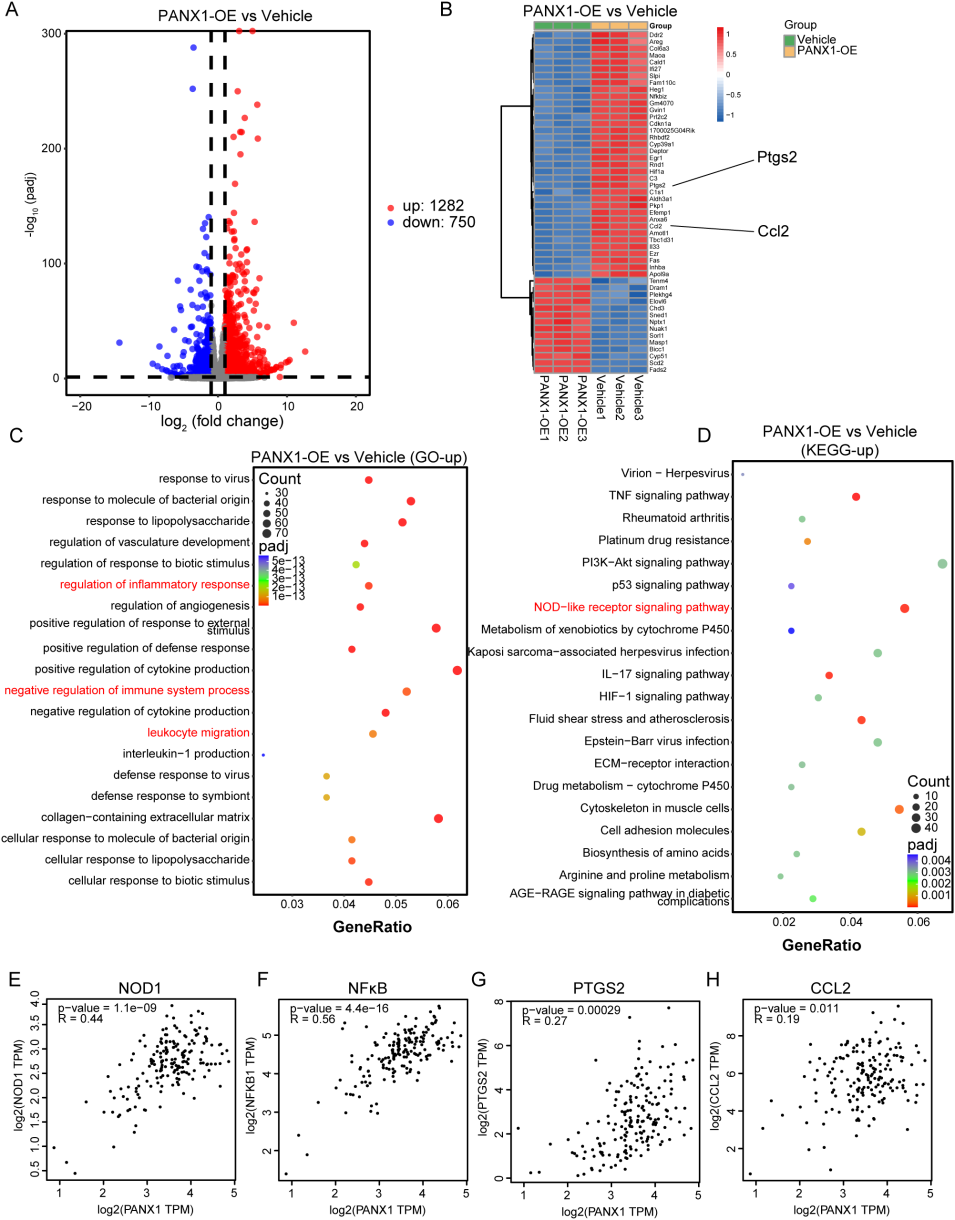
PANX1 was associated with immune regulation and NOD-like receptor signaling pathway. **(A)** The volcano map showed the DEGs between PANX1-OE and Vehicle group. **(B)** Heat maps showed the top 50 DEGs (e.g., Ptgs2 and Ccl2) between PANX1-OE and Vehicle group. **(C, D)** GO and KEGG analysis showed PANX1 was correlated with negative regulation of immune system process and NOD-like receptor signaling pathway, etc. **(E–H)** Pearson analysis shows the correlation between PANX1 and NOD1, NFκB, PTGS2, and CCL2.

**Figure 8:**
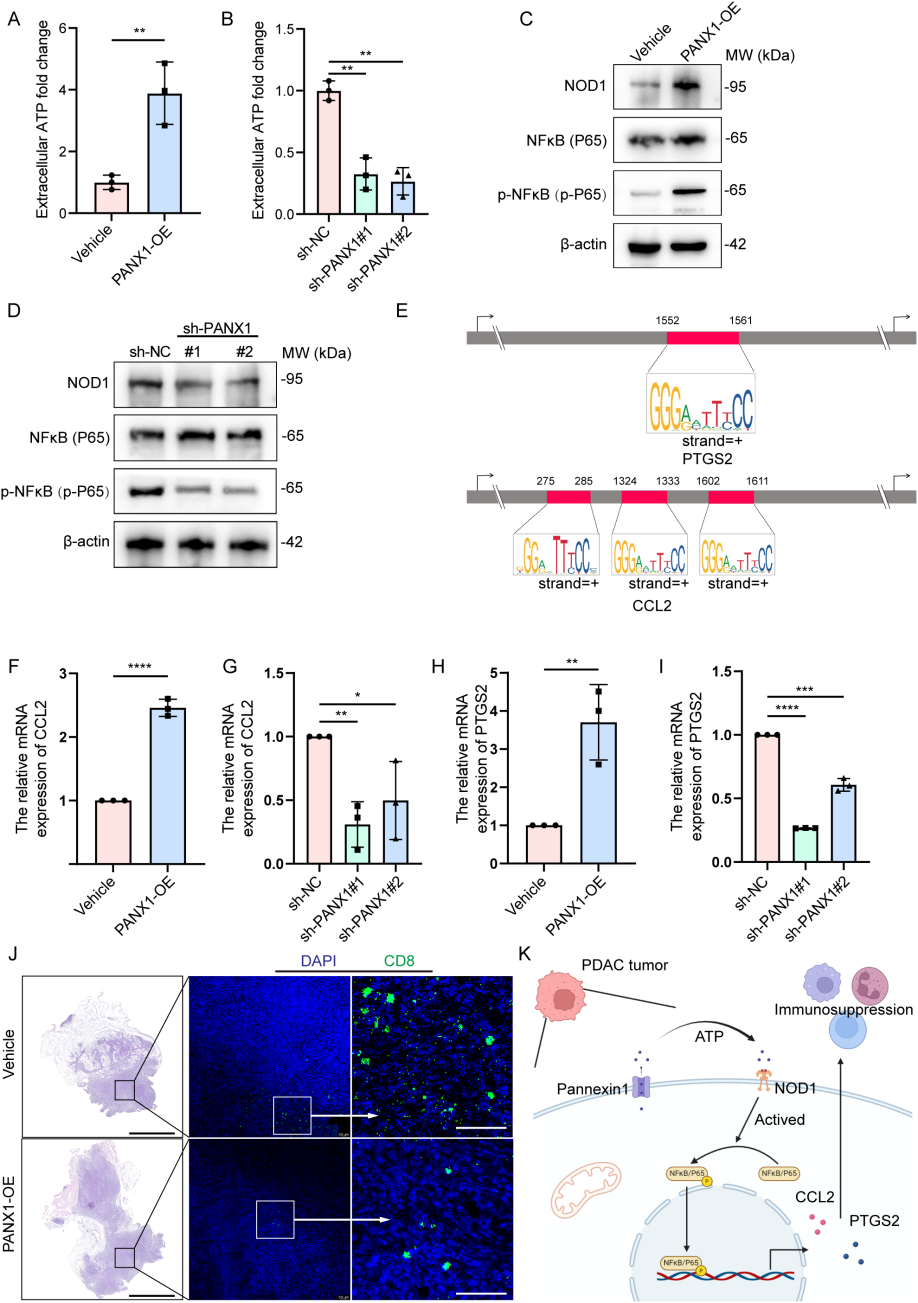
PANX1 promoted CCL2/PTGS2-induced immunosuppression via the ATP/NOD1/NFκB signaling pathway. **(A, B)** Overexpression of PANX 1 promoted and knock-down of PANX 1 inhibited extracellular ATP levels. **(C, D)** Overexpression of PANX 1 upregulated and knock-down of PANX 1 inhibited the protein level of NOD1 and Phosphorylated NFκB (p-P65). **(E)** Comprehensive survival analysis of patients with different tumor mutation loads and different prognostic risk. **(E)** Motif analysis showed the sites of transcription initiation regions where NFκB binds to PTGS2 (1552-1561) and CCL2 (275-285, 1324-1333,1602-1611). **(F–I)** Overexpression of PANX 1 upregulated and knock-down of PANX 1 inhibited the mRNA level of CCL2 and PTGS2. *P<0.05, **P<0.01, ***P<0.001, ****P<0.0001. **(J)** PANX 1 overexpression promotes CD8^+^ T cell infiltration *in vivo*, scale bar = 2 mm. **(K)** Research hypothesis chart.

To explore the mechanism of how PANX1 affects PDAC growth, we performed Bulk-RNA sequencing for PANX1-OE and Vehicle group in PDAC cell line Panc02. Among the DEGs (**Figures 7A, B**), PANX1-OE group showed upregulation of genes with known functions in immunosuppressive (Ccl2, Il33, Fas, Ptgs2, etc.). Enrichment analysis also showed that PANX1-OE group was positively correlated with immunomodulatory, NOD-like receptor signaling pathways (**Figures 7C, D**). Furthermore, PANX1 was positively correlated with genes (NOD1, NFκB, PTGS2, and CCL2) related to NOD-like signaling pathway (**Figures 7E–H**). Based on the function of PANX1 as a channel protein, extracellular ATP levels were measured. The PANX 1-overexressing-Panx02 cells significantly promoted ATP release, while PANX1 knockdown had opposite effects (**Figures 8A, B**). To investigate whether PANX1 could influence NOD-like signaling pathway, we examined changes in the expression of relevant genes (NOD1, NFκB, PTGS2, CCL2). The PANX1-overexressing-Panc02 cells promoted upregulation of NOD1 and Phosphorylated NFκB (p-P65), while PANX 1 knockdown had opposite effects (**Figures 8C, D**). Moreover, as a transcription factor, NFκB could bind the transcription initiation region of PTGS2 and CCL2 (**Figure 8E**). Therefore, PANX1 could affect the mRNA expression of CCL2 and PTGS2 accordingly (**Figures 8F–I**). The previous functional enrichment analysis found that PANX1 is associated with immune regulation, so we examined the expression of CD8^+^ T cells *in vivo* tissues of mice. PANX1-OE group had less CD8^+^ T cell infiltration (**Figure 8J**). A summary of the mechanism diagram is shown in the **Figure 8K**.

## 4 Discussion

Anti-cancer immunity and response to immune therapy of PDAC was influenced by the unique TME states of the tumors (17). Growing evidence shown that targeting the DAMPs of ICD process enhanced the specific immune response to pancreatic tumors (18, 19). DAMPs released by dead tumor cells, which are recognized by innate pattern recognition receptors, such as TLRs and NOD-like receptors (NLRs), thus activates tumor-specific immune responses, and then stimulates the long-term efficacy of anti-cancer drugs by directly killing cancer cells and anti-tumor immunity (20). In PDAC, DAMPs and their receptors are the potential predictors of immunotherapy response. Therefore, in this study, we stratified PDAC patients using the expression of DAMPs-related genes, and patients with poor prognosis had poor immune status. In addition, we established a prognostic risk signature based on DAMPs-related genes and predicted the PDAC patients who might benefit from immunotherapy.

Currently, the widespread use of high-throughput sequencing has revealed numerous novel therapeutic targets among cell subtypes within tumor tissues (21). We noted that, PDAC patients with high expression of DAMPs and its receptors have a poor prognosis which is consistent with previous studies (16). Subsequently, high levels of immune checkpoint molecules and HLA family genes were found in PDAC patients with poor prognosis. Unprecedented advances have been made in cancer treatment with the use of immune checkpoint blockade. Monoclonal antibodies are used to target immune checkpoint molecules by inhibiting cytotoxic T lymphocyte-associated antigen-4 (CTLA-4), programmed cell death protein 1 (PD-1) and programmed cell death 1 ligand 1 (PD-L1), together results in a shift mode of treatment of malignant tumors (22). Gebremeskel et al. found that oncolytic peptide could directly kill drug-resistant tumor cells, induce ICD, and produce a strong anti-tumor immune response (23). Therefore, immunotherapy has great prospects in the treatment of pancreatic cancer, and seeking immunotherapy-related markers is of guiding significance for the treatment of pancreatic cancer (24–27). These results suggested that ICD after inducing may enhance the therapeutic effect of ICIs.

Proverbially, PDAC is driven by KRAS mutation (28). Consistent with our findings, PDAC patients with poor prognosis had a higher mutation frequency of KRAS and TMB score. The activation of carcinogenic KRAS signal leads the tumorigenesis and tumor progression by the variation of TME. Dai et al. (29) determined that KRAS^G12D^, as a DAMPs, was released from tumor cells undergoing oxidative stress. The extracellular KRAS^G12D^ was packaged as an exocrine, and swallowed by macrophages through AGER. Next, they revealed that KRAS^G12D^ promotes macrophage polarization through STAT3-dependent oxidative activation of fatty acids. Therefore, patients with PDAC are likely to benefit from the prognostic signature of DAMPs-related genes.

Next, we found up-regulated DEGs were associated with immune signaling pathways by enrichment analysis. Moreover, the risk score was also related to immune cell infiltration. PDAC is immunosuppressive, characterized by rich fibrous stroma and suppressor immune cells including tumor-associated macrophages, Tregs and myeloid derived suppressor cells, and further limits the efficacy of chemotherapy or ICIs (30). It has been reported that immune cells and stromal cells accelerated tumor immune escape and progression (31). DAMPs-induced ICD provided a specific theoretical basis for anti-tumor immunotherapy due to its immunogenicity, immune activation in tumor and multiple tumor antigen release (32). He et al. (19) found that irreversible electroporation (IRE) induced ICD of tumor cells by increasing the synthesis and secretion of DAMPs. Moreover, IRE increased the release of HMGB1 and activated MAPK-p38 pathway by binding to AGER, and further resulted in the increased level of M1 markers in macrophages. These results indicated that DAMPs-related genes induced shift in the state of immune cells, which provided a new idea and strategy for combined immunotherapy of PDAC.

In our study, DAMPs prognostic signature was used to predict PDAC patients who were sensitive to gemcitabine and paclitaxel. Although gemcitabine is currently the standard choice for the treatment of PDAC, matrix components, including tumor associated macrophages, which is essential for transmitting gemcitabine resistance in PDAC cells (33, 34). Less than 15% of the patients had no progression within 6 months after receiving gemcitabine chemotherapy, and the efficacy of AG regimen (Albumin paclitaxel + Gemcitabine) was also not satisfactory (1). Therefore, our findings are expected to alleviate the limitations on the effectiveness of chemotherapy due to the drug resistance of PDAC cells.

Interestingly, five DAMPs-related genes (PANX1, TLR3, DDX58, P2RY2, and AGER), which constructed prognostic signature, also play roles in PDAC microenvironment. It is worth noting that PANX1 has the significance of further experimental verification. PANX1 is a non-selective ion channel protein predominantly located on the cell membrane, facilitating the passage of small molecules like ATP, glutamate, and GABA. This function enables intercellular signal transmission. Metabolism plays a key role in inflammation (35). In this study, we conducted *in vivo* and *in vitro* experiments along with RNA sequencing to substantiate the role of PANX1 in eliciting the expression of CCL2 and PTGS2 via the ATP release to active NOD1/NFκB signaling pathway. This mechanism contributes to immunosuppression and the advancement of PDAC. Consistent with our results, overexpression of PANX1 was associated with poor prognosis of PDAC and increased infiltration of immunocytes, such as tumor associated fibroblasts, macrophages and neutrophils (36). The study reveals that in NOD1, the γ-phosphate of ATP is positioned towards the central nucleotide binding cavity, resembling NLRC4 (37). The important signaling pathways that affect the occurrence and progression of diseases are becoming clearer (38, 39). Moreover, NFκB, being a classical downstream signaling pathway of NOD1, has the ability to trigger the transcription and expression of immunosuppressive molecules like PTGS2 (40) and CCL2 (7). Taken together, these studies shown that the prognostic signature constructed by DAMPs-related genes (PANX1) had great potential in the progress and treatment of PDAC.

However, our research also has limitations: 1) Due to the limitation of sample size, the prognostic signature was not accurately predicting PDAC patients who respond to immunotherapy. Although TCGA train set and GEO validation set were used for analyze, a large number of PDAC samples for validation in the future remains needed. 2) *In vivo* and *in vitro* experiments investigating PANX1 are still ongoing, including the absence of targeted PANX1 combined immunotherapy experiments. For example, anti-panx1 antibody combined with ICB (αPD1, αPDL1, αCTLA4) is used to treat pancreatic cancer (41).

## 5 Conclusion

In conclusion, we comprehensively analyzed the expression profiles and mutation information from multiple databases, and constructed a DAMPs prognostic signature to predict the survival prognosis and immunotherapy responsiveness of patients with PDAC. In particular, PANX1 plays a key role in immune regulation and tumor progression in PDAC.

## Data Availability

The datasets presented in this study can be found in online repositories. The names of the repository/repositories and accession number(s) can be found below: https://bigd.big.ac.cn/gsa/browse/CRA019259, GSA: CRA019259.

## Supplementary material

Supplementary Figure 1 Protein-protein interaction of DAMPs-related genes in PDAC.

Supplementary Figure 2 DEGs between two DAMPs subtypes in Pancreatic carcinoma. **(A)** Heatmap of DEGs between two DAMPs subtypes of PDAC. **(B)** Volcanic map of DEGs between two DAMPs subtypes of PDAC. **(C–E)** Enrichment analysis of up-regulated DEGs between two DAMPs subtypes of PDAC.

Supplementary Figure 3 Gene mutation and tumor immunity in different DAMPs subtypes of PDAC. **(A)** Frequency of gene mutation in different DAMPs subtypes of PDAC. **(B–E)** Relationship between ICD genotyping and TME in PDAC. **(F)** Relationship between ICD genotyping and immune cell content in pancreatic carcinoma.

Supplementary Figure 4 DAMPs prognostic signature was used to predict the survival rate of patients with PDAC. **(A)** The C index of risk score and various clinical features. **(B)** Nomogram is used to calculate the survival rate of patients with PDAC using risk scores and various clinical features. **(C)** The calibration curve validated the predictive ability of the nomogram. **(D)** The relationship between risk score and tumor immunity.

Supplementary Figure 5 Gene expression level of DAMPs prognostic signature in PDAC. **(A)** Immunohistochemical staining of the genes (PANX1, TLR3, DDX58, P2RY2, and AGER), which constructed the DAMPs prognostic signature. **(B)** TLR3, DDX58, P2RY2, and AGER was upregulated in PDAC cancer. **P<*0.05. **(C, D)** TLR3, DDX58, and P2RY2 were correlated with poor overall survival (OS) and disease-free survival (DFS), while AGER was associated with favorable OS and DFS outcomes.
