# Supplementary figures and images for: DAMPs prognostic signature predicts tumor immunotherapy, and identifies immunosuppressive mechanism of pannexin 1 channels in pancreatic ductal adenocarcinoma
Source: Front Immunol. 2025 Jan 15;15:1516457. doi: 10.3389/fimmu.2024.1516457 (PMC11775746; doi:10.3389/fimmu.2024.1516457)

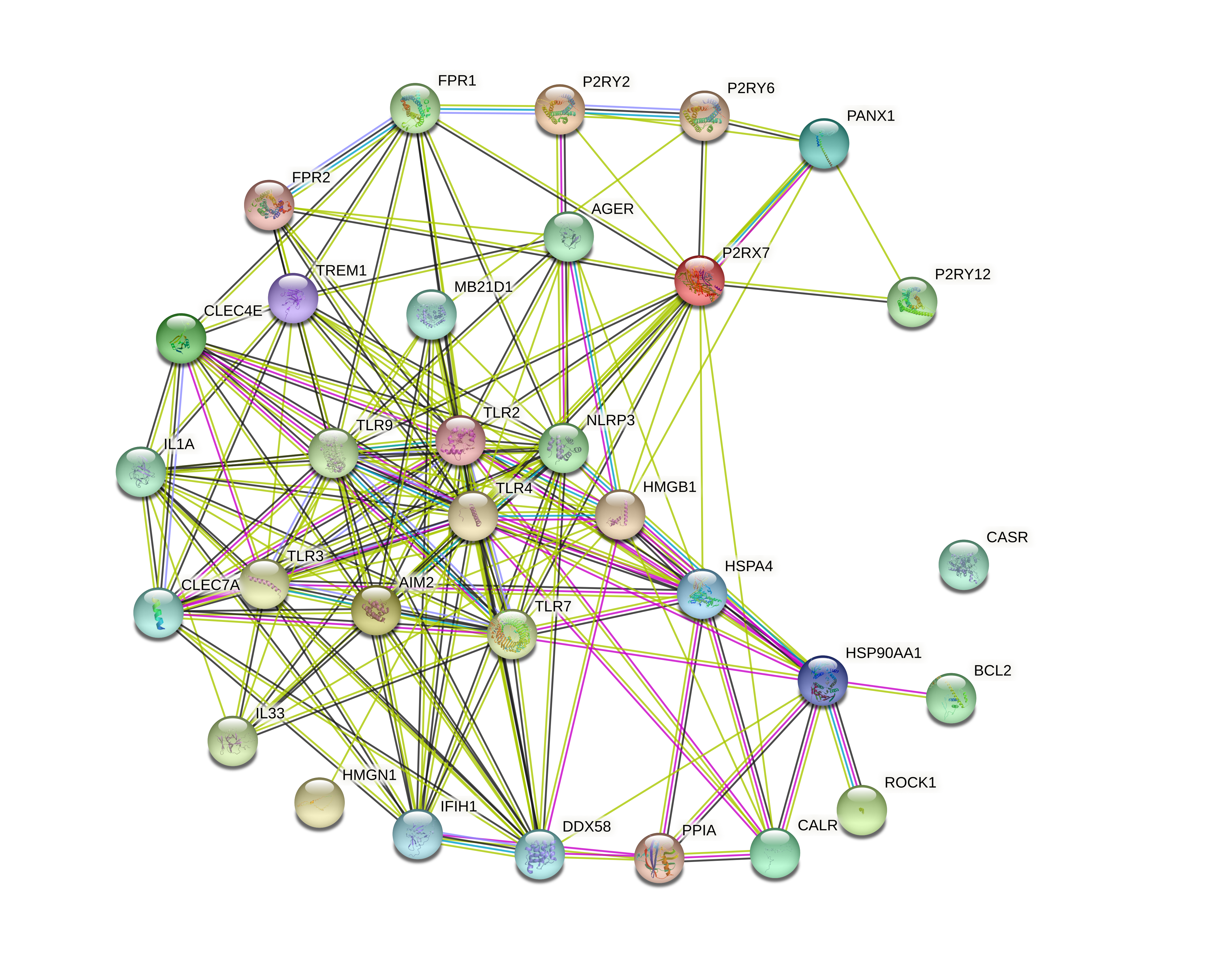

Supplement: Supplementary Figure 1 — Protein-protein interaction of DAMPs-related genes in PDAC. [file Image1.png]

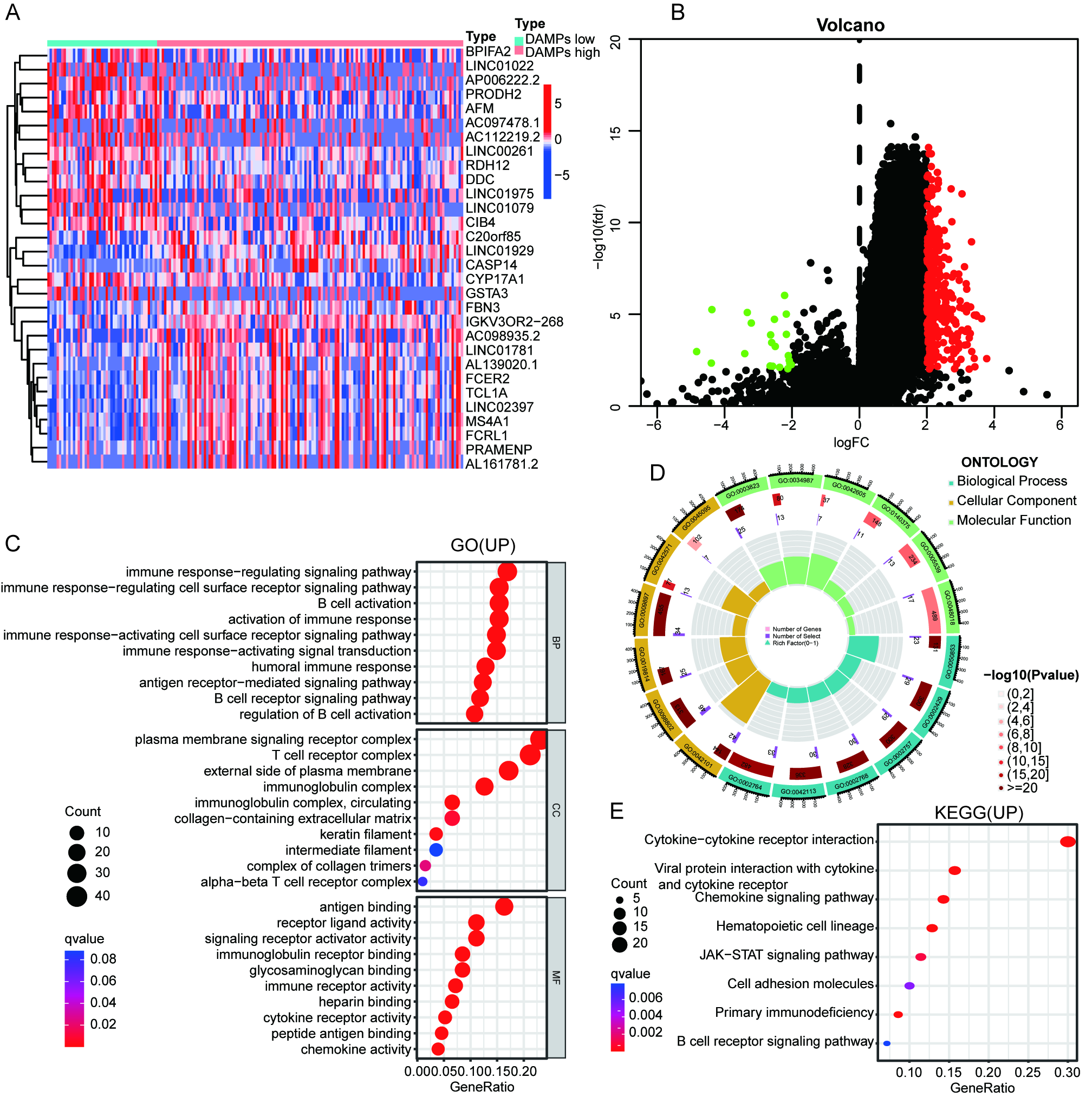

Supplement: Supplementary Figure 2 — DEGs between two DAMPs subtypes in Pancreatic carcinoma. (A) Heatmap of DEGs between two DAMPs subtypes of PDAC. (B) Volcanic map of DEGs between two DAMPs subtypes of PDAC. (C–E) Enrichment analysis of up-regulated DEGs between two DAMPs subtypes of PDAC. [file Image2.tif]

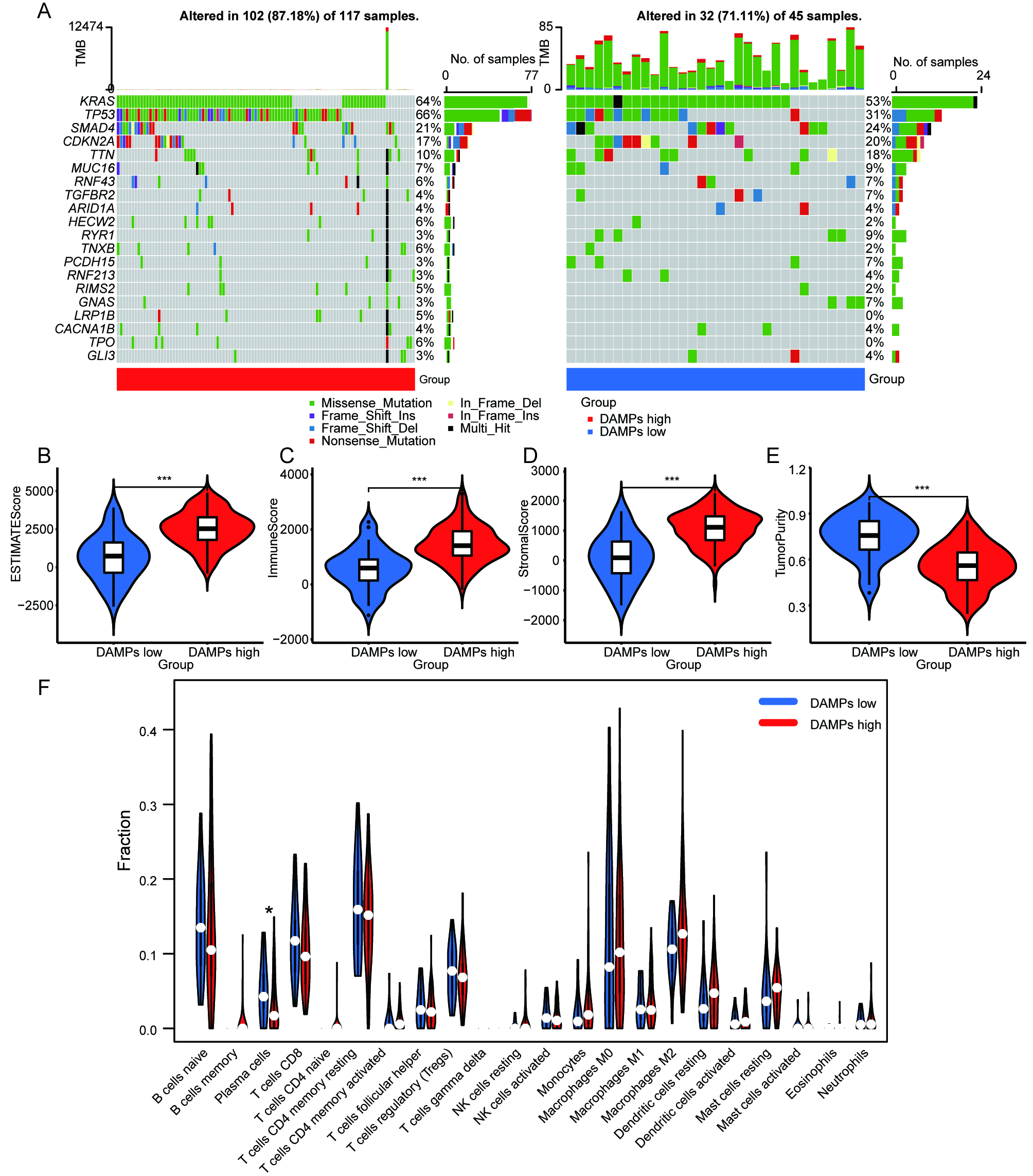

Supplement: Supplementary Figure 3 — Gene mutation and tumor immunity in different DAMPs subtypes of PDAC. (A) Frequency of gene mutation in different DAMPs subtypes of PDAC. (B–E) Relationship between ICD genotyping and TME in PDAC. (F) Relationship between ICD genotyping and immune cell content in pancreatic carcinoma. [file Image3.tif]

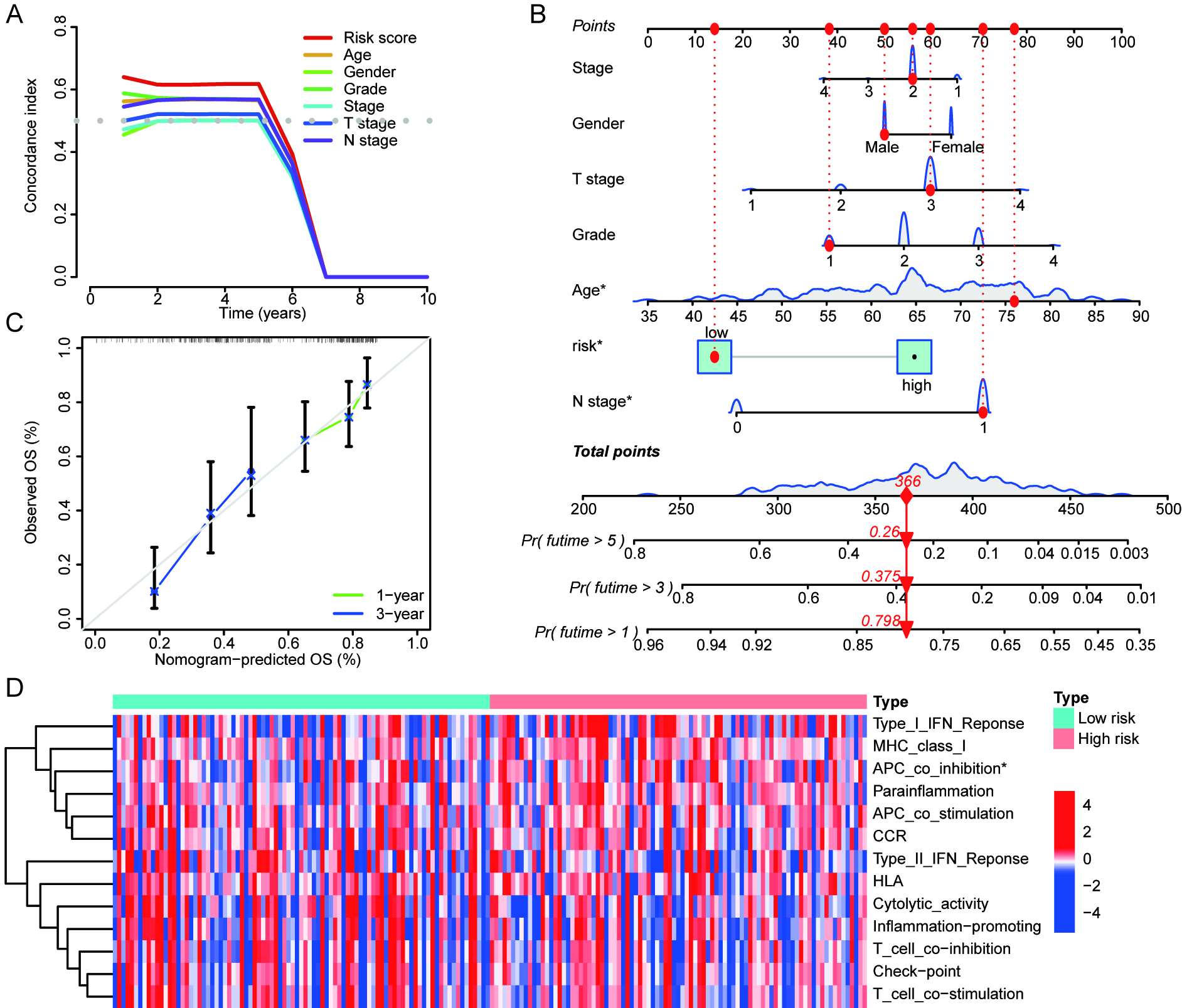

Supplement: Supplementary Figure 4 — DAMPs prognostic signature was used to predict the survival rate of patients with PDAC. (A) The C index of risk score and various clinical features. (B) Nomogram is used to calculate the survival rate of patients with PDAC using risk scores and various clinical features. (C) The calibration curve validated the predictive ability of the nomogram. (D) The relationship between risk score and tumor immunity. [file Image4.tif]

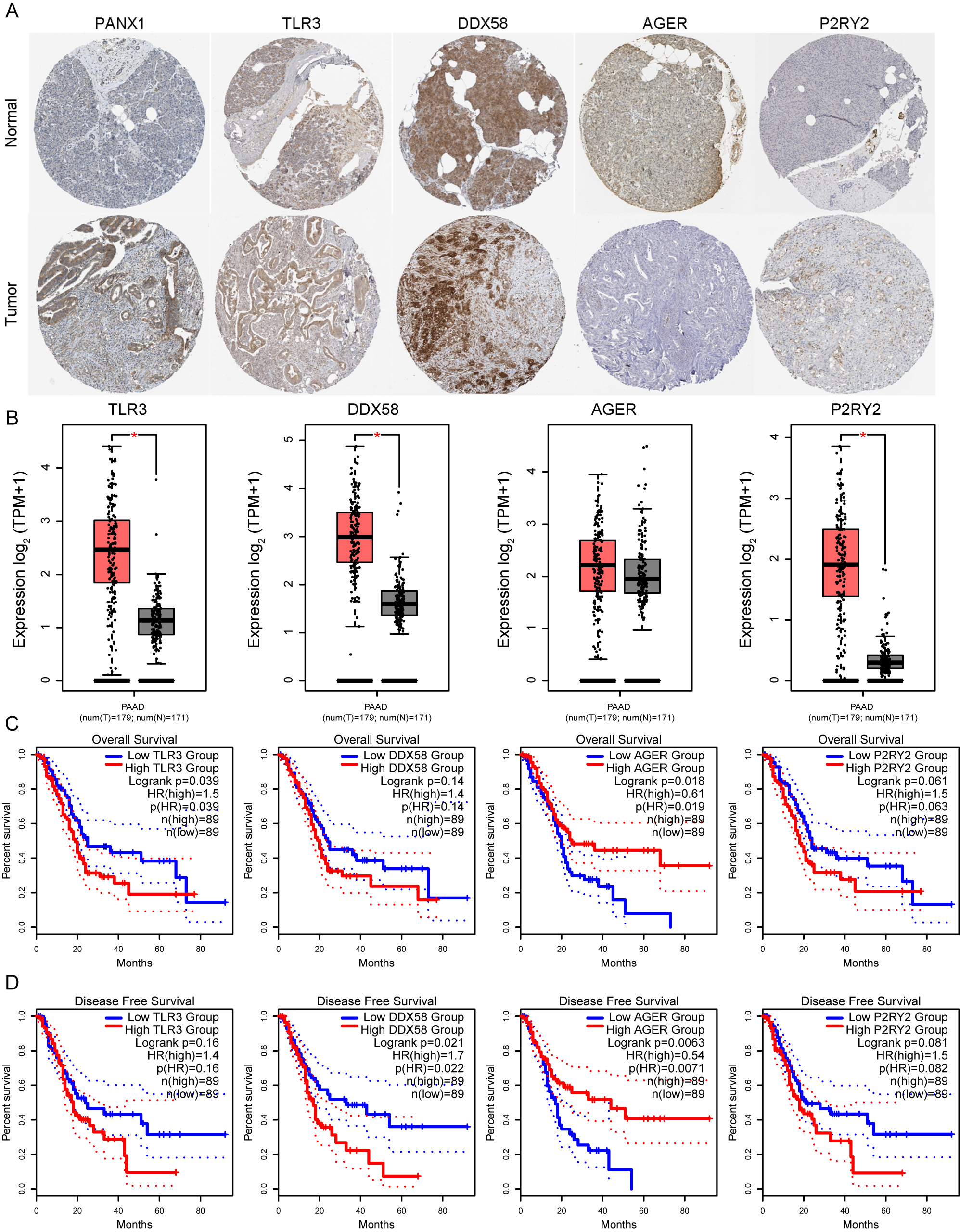

Supplement: Supplementary Figure 5 — Gene expression level of DAMPs prognostic signature in PDAC. (A) Immunohistochemical staining of the genes (PANX1, TLR3, DDX58, P2RY2, and AGER), which constructed the DAMPs prognostic signature. (B) TLR3, DDX58, P2RY2, and AGER was upregulated in PDAC cancer. *P<0.05. (C, D) TLR3, DDX58, and P2RY2 were correlated with poor overall survival (OS) and disease-free survival (DFS), while AGER was associated with favorable OS and DFS outcomes. [file Image5.tif]
