# Supplementary material for: DAMPs prognostic signature predicts tumor immunotherapy, and identifies immunosuppressive mechanism of pannexin 1 channels in pancreatic ductal adenocarcinoma
Source: Front Immunol. 2025 Jan 15;15:1516457. doi: 10.3389/fimmu.2024.1516457 (PMC11775746; doi:10.3389/fimmu.2024.1516457)
Supplement: Supplementary file 6 [file Table1.docx]

Table S1 The Primers and Antibodies:

| Panx1-F | GGTCTACCTCTGCTGCTCATCT |
| --- | --- |
| Panx1-R | CCAGCAGTATGAATCCACAAAGG |
| Ptgs2-F | TTGCATTCTTTGCCCAGCAC |
| Ptgs2-R | ACCGTAGATGCTCAGGGACT |
| Ccl2-F | AGCAGCAAGTGTCCCAAAGA |
| Ccl2-R | GGTGTCTGGGGAAAGCTAGG |
| Gapdh-F | CATCACTGCCACCCAGAAGACTG |
| Gapdh-R | ATGCCAGTGAGCTTCCCGTTCAG |
| Anti-PANX1 | Huaan bio#JE38-97 |
| Anti-NOD1 | CST#3545 |
| Anti-NFκB (P65) | CST#8242 |
| Anti-p-NFκB (P65) | CST#3033 |
| Anti-CD8 | Abcam# ab237709 |
| Anti-β-actin | CST# 4967 |
| Anti-mouse IgG, HRP-linked Antibody | CST #7076 |
| Anti-rabbit IgG, HRP-linked Antibody | CST #7074 |
| Anti-rabbit IgG (H+L), F(ab')2 Fragment (Alexa Fluor® 488 Conjugate) | CST #4412 |
