# Supplementary material for: DAMPs prognostic signature predicts tumor immunotherapy, and identifies immunosuppressive mechanism of pannexin 1 channels in pancreatic ductal adenocarcinoma
Source: Front Immunol. 2025 Jan 15;15:1516457. doi: 10.3389/fimmu.2024.1516457 (PMC11775746; doi:10.3389/fimmu.2024.1516457)
Supplement: Supplementary file 7 [file Table2.docx]

attach files: https://www.jianguoyun.com/p/DQD0iXsQidSBDRjzqNwFIAA
